# Effect of Sheep’s Milk Composition on Strength and Syneresis of Rennet-Induced Milk Gel During Lactation

**DOI:** 10.17113/ftb.57.03.19.6218

**Published:** 2019-09

**Authors:** Ante Rako, Milna Tudor Kalit, Samir Kalit

**Affiliations:** 1 Institute for Adriatic Crops and Karst Reclamation, Put Duilova 11, HR-21000 Split, Croatia; 2 University of Zagreb, Faculty of Agriculture, Department of Dairy Science, Svetošimunska 25, HR-10000 Zagreb, Croatia

**Keywords:** sheep’s milk, lactation, syneresis, gel strength

## Abstract

This study investigates the effects of raw sheep’s milk composition on the strength and syneresis of obtained gels throughout the lactation. Casein, fat and total solids content, as well as ionic calcium mass fraction significantly (p<0.05) increased during lactation. As lactation progressed, milk formed firmer gel with higher syneresis ability. Increasing casein to fat ratio in sheep’s milk significantly increased (p<0.05) gel strength and syneresis. On the other hand, gel strength and syneresis were significantly reduced as a result of increased fat content in sheep’s milk. Ionic calcium mass fraction affected gel strength but not syneresis. Neither gel strength nor syneresis were affected by casein and urea content or by somatic cell count in sheep’s milk. Correlation coefficients between milk components and gel strength, as well as syneresis, were significant (p<0.01, p<0.05) but never higher than 0.35.

## INTRODUCTION

The rennet coagulation of milk involves the conversion of milk from a colloidal dispersion of stable casein micelles to a network of aggregated paracasein micelles in a continuous phase entrapping fat and moisture in its pores (*1*). The essential step in cheese manufacturing is obtaining a curd of proper strength to withstand mechanical cutting in cheese vats without shattering into fines, which would result in casein and fat loss into whey (*2*). In addition, curd at an appropriate firmness must also be able to expel the amount of whey through controlled syneresis. This enables retention of the desired level of calcium phosphate in the curd through controlled production using lactic acid bacteria and incorporation of the desired amount of salt into the final cheese (*2*).

Numerous factors such as stage of lactation, pretreatment of milk, addition of calcium, rennet concentration and renneting temperature of milk affect the curd firmness to various extents but milk composition is of primary importance (*1*). In support of the aforementioned claim, Grandison *et al.* (*3*) reported that even relatively small changes in its composition radically affect the coagulation properties of milk. It is well recognized that the degree of recovery of casein and fat from milk into the curd is the highest in comparison to the other milk components and consequently contributes more significantly to its rheological properties. From a rheological standpoint, casein is the main constituent that builds the structure of continuous paracasein framework and gives, when intact, the elastic and solid character of the curd while the contribution of fat to the rheological properties of the paracasein framework primarily depends on renneting temperature (*4*). Therefore, many studies of the effect of milk composition on its coagulation properties have been focused on casein and fat content (*5*).

Gel strength is also greatly affected both by the calcium content and pH value of milk. The removal of caseinomacropeptide by rennet leads to exposure of negatively charged amino acid residues of calcium-sensitive caseins to calcium ions (*6*). The free ionic calcium shields the negatively charged amino acid residues, enhancing the aggregation of casein micelles, leading to more interactions within the gel network and thereby obtaining higher curd firmness (*6*). On the other hand, the strength of a rennet gel also depends on the volume and homogeneity of the calcium phosphate paracasein network, which determines the number of stress-bearing strands per unit area of the gel (*7*). The interior of a casein micelle is composed of calcium-sensitive caseins cemented into the micelle structure by strong linkages to the calcium phosphate and cross-linked by hydrophobic bonding between the casein chains (*7*). Van Hooydonk *et al.* (*8*) reported solubilization of calcium phosphate as the most important effect of lowering the pH of milk. The removal of calcium phosphate causes reduction in the number of cross-links within the strands of the casein gel network, resulting in a decrease in gel firmness (*6*, *9*). Since the colloidal form of calcium phosphate is positively charged, its removal leads to an increase of the already net negatively charged casein, which results in more electrostatic repulsion between caseinate building blocks and a lower gel firmness (*6*).

Casein micelles are reactive over their whole surface and, after the gel has been formed, they have the ability to form more new bonds among paracasein micelles. This leads to additional reduction of space within the paracasein network, which causes formation of a more compact gel structure and expulsion of the whey from the gel (*10*). The effect of milk composition on syneresis is still under debate as it has been reported as marginal by some (*1*) and as of great influence by others (*11*).

As far as the authors know, the combined effect of physicochemical characteristics of sheep’s milk and lactation stage has never been studied in relation to strength and syneresis of rennet-induced sheep’s milk gel. Pellegrini *et al.* (*12*) studied the effect of physicochemical characteristics of sheep’s milk on the firmness but not on the syneresis of rennet-induced milk gel. Storry *et al.* (*13*) also conducted research to determine physicochemical composition and coagulation properties of renneted milk from different species of ruminants but their experiments were performed on just four samples of sheep’s milk. Bornaz *et al.* (*11*) compared physicochemical characteristics and renneting properties of camel’s milk with sheep’s, goat’s and cow’s milk obtained in Tunisia without focusing on the influence of lactation. The experiments regarding the effect of physicochemical characteristics of sheep’s milk on the strength and syneresis of rennet-induced milk gel have been conducted to a lesser extent than those regarding cow’s milk. Strength and syneresis of rennet-induced milk gel are important factors in cheese making since they affect parameters such as cheese yield, moisture content and textural attributes of cheese. These cheese attributes are important for production cost-effectiveness in cheese industry (*14*). Therefore, the aim of the study is to assess the influence of physicochemical characteristics of sheep’s milk on strength and syneresis of rennet-induced milk gel.

## MATERIALS AND METHODS

### Milk sampling and analysis

A total of 42 bulk milk samples were collected and analysed. The study was carried out during two successive years in flocks of Dalmatian Pramenka sheep on three family farms. Two farms were located on the island of Brač, Croatia. The first farm is situated at an altitude of 207 m above sea level in the area of Supetar. The second farm is in the inner part of the island in the area of Pražnice at an altitude of 437 m above sea level. The third farm is in the area of Maovice in inland part of Dalmatia on the north slopes of mountain Svilaja, at an altitude of 640 m above sea level. Lactation period on the first farm was from mid-February until mid-June and lasted on average 120 days. On the second farm lactation started at the beginning of May and ended in mid-July, and lasted on average 75 days. On the third farm, sheep were milked from the first half of April until the end of the first week of August, and during 126 days of lactation the sheep mostly grazed on pastures of mountain Svilaja at an altitude of 1000–1100 m above sea level. The lactation period on each farm was divided into three equally long stages: early, middle and late. The lactation stages on the first farm lasted 40 days each, on the second farm 25 days each and on the third farm 42 days each. Once lambing started on each farm, sheep that were lambed within two weeks were considered. During this research, 100, 50 and 300 sheep were milked twice a day on the first, second and third farm, respectively. Throughout the entire lactation, 1500 mL of raw sheep’s milk samples were collected every 14 days from cooling tanks, two hours after the morning milking. Milk samples represented the mixture of evening and morning milking. Six milk samples from early lactation, six milk samples from middle lactation and four milk samples from late lactation period were taken from the first farm. Since the lactation on the second farm was shorter than on the other two, early and middle lactation included four milk samples each, while the late lactation included two milk samples. The number of milk samples taken from the third farm from each lactation stage was the same as from the first farm. In total, the number of milk samples collected from the first farm was 16, from the second 10 and from the third 16. Milk samples were transported in a portable refrigerator to a laboratory and stored at 4 °C until used in the experiment (within the next 24 h). The content of protein and fat were analysed by infrared spectroscopy using a MilkoScan FT 120 (Foss, Hillerød, Denmark) according to ISO 9622:2013 (*15*). The method is based on the fact that the specific chemical bonds present in protein and fat molecules (as well as other macromolecules in milk) absorb a different amount of IR radiation of defined wavelengths. Based on the absorbed amount of IR radiation, the instrument calculates the protein and fat content. Determination of casein nitrogen content in milk was carried out by block digestion method according to validated modification of ISO 8968-1:2014 (*16*) using a Kjeltec 2300 Tecator (Foss). Milk is digested with a strong acid so that it releases nitrogen which is determined by titration. The amount of casein present is then calculated from the nitrogen concentration of the milk. Urea concentration was determined by enzymatic method using difference in pH according to ISO 14637:2004 (*17*). The measure is based on an enzymatic reaction, the urea hydrolysis by urease, which causes a pH variation directly proportional to the urea concentration in the milk sample. Potentiometric method using the ion selective electrode perfectION^TM^ comb Ca^2+^ (Mettler Toledo, Greifensee, Switzerland) measured ionic calcium mass fraction while pH was determined by InLab Solids Pro ISM electrode (Mettler Toledo) according to the manufacturer’s manual (*18*). Prior to measurements, ion selective electrode was calibrated with two standards that bracket the expected sample range and differ in concentration by a factor of ten. Somatic cell count was determined by fluoro-optic-electronic method using a Fossomatic™ Minor (Foss) according to ISO 13366-2:2006 (*19*). The DNA of somatic cells was stained by propidium iodide and then counted by charge-coupled device (CCD) of the instrument. The total bacterial count was determined by flow cytometry method using a BactoScan FC73700 (Foss) according to ISO 21187:2004 (*20*). The bacterial DNA was stained with ethidium bromide and then counted by the optical part of the instrument. The bacterial count (viable and non-viable bacteria) was then converted in the number of viable bacteria (CFU) according to the instructions given in the standard (*20*). Each analysis was performed in duplicate.

### Measurement of gel strength and syneresis

Syneresis and gel strength of the obtained curd were measured at the Institute of Adriatic Crops and Karst Reclamation in Split, Croatia. A laboratory beaker was filled with 1000 mL of milk, left in a water bath at 32 °C and allowed to equilibrate. After the equilibration, rennet (CHY-MAX extra; Chr. Hansen, Hørsholm, Denmark) was added to the milk (0.12 g/kg) following the manufacturer’s recommendation. A volume of 600 mL of milk was removed from the laboratory beaker immediately after rennet addition and evenly distributed into six glass beakers (100 mL each) for the purpose of measuring gel strength. The glass beakers were left in a water bath at 32 °C to coagulate over different coagulation times (45, 60 and 75 min). Two replicates were analysed for each coagulation time. After expiration of coagulation time, gels within the six beakers were subjected to gel strength measurement at room temperature (25 °C) by texture analyser (TA Plus; Lloyd Instruments, Fareham, UK) equipped with a 500 N load cell (XLC -0500-A1; Lloyd Instruments) and cylindrical probe (FG/CY3; Lloyd Instruments). The experimental data were analysed by Nexygen Plus 3 software (*21*). A cylinder probe was used to penetrate the curd in the glass beaker at crosshead speed of 10 mm/min until it reached a depth of 20 mm. Gel strength was evaluated as a maximum load applied during the test. The remaining milk (400 mL) was distributed into six centrifuge tubes (30 g for each) for the purpose of measuring syneresis. After the formation of the gel in centrifuge tubes in the water bath (32 °C) at the specified coagulation time (45, 60 and 75 min), the gel was centrifuged at 3630 relative centrifugal force (RCF) for 15 min (Universal 320 centrifuge; Hettich, Tuttlingen, Germany) and the expelled whey was poured off and weighed. The syneresis was determined as grams of expelled whey per 100 g of gel. Two replicates were analysed for each coagulation time.

### Statistical analysis

Statistical evaluations of the results were conducted using SPSS v. 21 (*22*). One-way ANOVA and general linear model (GLM) procedure were used to determine the effect of the stage of lactation on sheep’s milk composition. Repeated measures ANOVA using GLM procedure was performed to determine the effect of milk composition during three stages of lactation on the strength and syneresis of gels obtained at three different coagulation times. The amounts of casein and fat, urea and ionic calcium mass fraction, as well as casein to fat mass ratio and somatic cell count were divided into three groups. The two between-subjects factors were milk components and lactation stage. The within-subjects factor was coagulation time. No significant interactions were found between these variables. When significant effects were found, the least significant difference (LSD) test was used to evaluate the differences between treatment mean values. Significance was indicated by p<0.05. Pearson’s coefficients of correlation were calculated for estimation of relationships among variables.

## RESULTS AND DISCUSSION

### Lactation related changes in milk constituents and gel characteristics

Modulation in milk constituents as well as gel strength and syneresis throughout lactation was extensively studied and well-documented in numerous studies, and therefore it was presented but not discussed in this research. Composition of raw sheep’s milk throughout lactation is shown in Table 1. Casein content significantly (p<0.01) increased from 4.49 at the beginning to 4.63 in the middle and 4.89 g/100 g in the late lactation stage. The protein content followed a similar trend to that of casein (5.7, 5.8 and 6.3 g/100 g in early, mid and late lactation, respectively). The LSD test showed that significant increase in the content of protein and casein occurred in late compared to early and middle stages of the lactation period (Table 1). It was also observed that protein (correlation coefficient R=0.48) and casein (R=0.51) contents significantly (p<0.01) correlate with the stage of lactation (Table 2). Along with the increase of protein and casein contents, a significant (p<0.05) increase in total solids and fat, as well as ionic calcium mass fraction were observed in sheep’s milk with advancement of lactation (Table 1). The lowest content of total solids in milk was observed in early lactation (18.6 g/100 g) and was followed by a moderate increase in mid lactation (19.0 g/100 g) reaching a markedly higher value at the end of lactation (19.8 g/100 g). The contents of fat gradually increased during lactation (7.5, 7.8 and 8.3 g/100 g in early, mid and late lactation, respectively). Furthermore, contents of total solids (R=0.43) and fat (R=0.35) significantly (p<0.05) correlated with stage of lactation (Table 2). Ionic calcium had the lowest mass fraction (10.9 mg/100 g) in sheep’s milk at the beginning of lactation. Its mass fraction continued to increase gradually (11.4 mg/100 g) in the middle of lactation, reaching a peak (12.8 mg/100 g) towards the end of lactation (Table 1). In addition, a statistically significantly positive correlation coefficient (R=0.46; p<0.01) was found between ionic calcium mass fraction and stage of lactation (Table 2). Urea concentration and somatic cell count in sheep’s milk remained relatively constant while bacterial count showed significant (p<0.05) increase as lactation progressed (data not shown).

**Table 1:** Contents of total solids, protein, casein, fat and ionic calcium in sheep’s milk at different stages of lactation

Component	Stage of lactation			p-value
Early (*N*=16)	Middle (*N*=16)	Late (*N*=10)
*w*(total solids)/(g/100 g)	(18.6±0.3)^a^	(19.1±0.3)^ab^	(19.8±0.3)^b^	<0.05
*w*(protein)/(g/100 g)	(5.7±0.1)^a^	(5.8±0.1)^a^	(6.3±0.1)^b^	<0.01
*w*(casein)/(g/100 g)	(4.49±0.08)^a^	(4.63±0.07)^a^	(4.89±0.08)^b^	<0.01
*w*(fat)/(g/100 g)	(7.5±0.3)^a^	(7.8±0.2)^ab^	(8.3±0.3)^b^	<0.05
*w*(Ca^2+^)/(mg/100 g)	(10.9±0.4)^a^	(11.4±0.4)^a^	(12.8±0.5)^b^	<0.05

Data are expressed as mean value±standard error. Mean values within a row with different letter in superscript differ significantly (p<0.05). *N*=number of samples

**Table 2:** The correlation coefficients between physicochemical composition of sheep’s milk, stage of lactation and characteristics of rennet-induced milk gel

Parameter	Stage of lactation	Parameter	Gel strength	Parameter	Syneresis	Parameter	Casein
Gel strength	0.28**	Casein to fat ratio	0.33**	Casein to fat ratio	0.25**	Fat	0.74**
Syneresis	0.24*	Total solids	-0.25*	Fat	-0.32**		
Protein	0.48**	Fat	-0.26**				
Casein	0.51**	Ionic calcium	0.25*				
Total solids	0.43*	Syneresis	0.22*				
Fat	0.35*						
Ionic calcium	0.46**						

Mean values followed by different letters in superscript differ significantly, *(p<0.05), **(p<0.01)

**Fig. 1:**
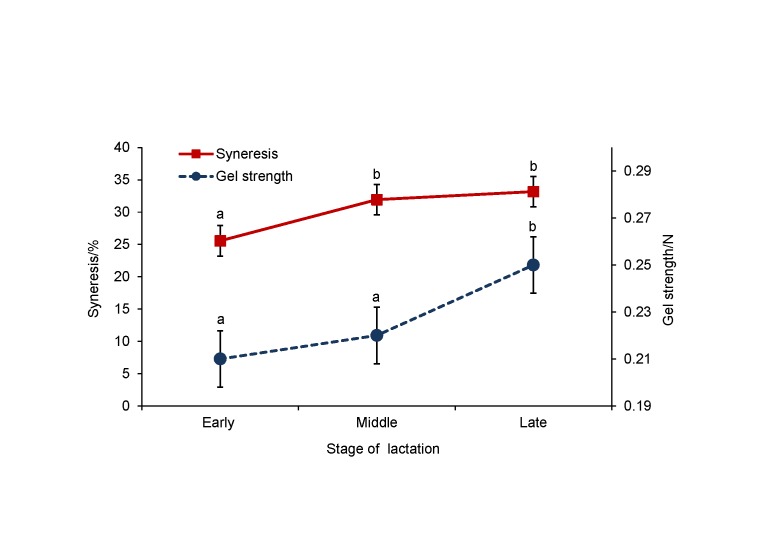
Gel strength and syneresis of rennet-induced milk gel at different stages of lactation

Both gel strength and syneresis significantly (p<0.05) increased during lactation (Fig. 1). According to the LSD test, the gel obtained from late lactation milk showed significantly (p<0.05) higher strength value (0.25 N) than the gels obtained from early (0.21 N) and mid (0.22 N) lactation milk. The lowest syneresis ability of rennet-induced milk gel was observed in early lactation (25.57%). The marked increase in syneresis was found in the middle stage of lactation (31.95%) and, although moderately, syneresis increased towards the end of lactation period (33.19%). Gel strength (R=0.28; p<0.01) and syneresis (R=0.24; p<0.05) were significantly correlated with stage of lactation (Table 2). However, a weak but significant (R=0.20; p<0.05) correlation was found between syneresis and gel strength (Table 2).

### Effect of milk constituents on gel strength and syneresis

The effects of casein and fat content, as well as casein to fat mass ratio, on syneresis ability and gel strength are presented in Table 3. The results of this research show that gel strength was not affected by casein content in sheep’s milk. This finding is not in agreement with Bornaz *et al.* (*11*), who reported a negative relationship between casein content and gel strength. These results are surprising since the majority of studies show a positive relationship between these parameters, indicating casein as one of the major factors contributing to gel firmness (*5*, *12*, *13*). The gel strength was quite similar regardless of the increase of casein content in sheep’s milk (Table 3) indicating that casein is not the sole compositional factor which affects gel strength (*13*). A possible explanation for the increase in casein content without changes in the strength of the obtained gels could be associated with parallel and significant (R=0.74, p<0.01) increase in fat content in sheep’s milk (Table 2). Casein forms the continuous paracasein sponge-like network that in relation to other milk ingredients (water, lactose, whey proteins) largely occludes the fat (*4*). As reported previously (*1*), the recovery of fat in cheese increased with increasing protein content in milk. Rheological properties of the paracasein framework depend on the temperature of occluded milk fat because it influences the ratio of solid to liquid fat. The proportion of liquid fat increases above 15 **°**C (*4*). Since the strength of milk gels in our study was measured at room temperature of 25 **°**C, it could be assumed that most of the fat occluded within the paracasein matrix in this study was liquid and tended to reduce rigidity of the casein network. The liquid fat acts as a lubricant on fracture surfaces of the casein matrix and thereby reduces the stress required to fracture the matrix (*1*). Casein content in sheep’s milk did not affect syneresis of the obtained gels (Table 3). Bornaz *et al.* (*11*) and Daviau *et al.* (*23*) found that the increase in casein content in milk reduced gel syneresis capacity as a consequence of low porosity and permeability of gels obtained from sheep’s milk. In contrast to these results, Jaramillo *et al.* (*24*) reported a positive but low correlation between increasing casein content in sheep’s milk and gel syneresis ability.

**Table 3:** Strength and syneresis of rennet-induced milk gel affected by casein and fat contents, casein to fat mass ratio, ionic calcium mass fraction, pH and somatic cell count in sheep’s milk

Component	*N*	Strength/N	Syneresis/%	p-value	
Strength	Syneresis
*w*(casein)/(g/100 g)					
4.08-4.53	13	0.23±0.01	30.9±3.9	n.s.	n.s.
4.54-4.98	15	0.22±0.01	24.4±2.8		
4.99-5.45	14	0.23±0.02	26.4±6.0		
*w*(fat)/(g/100 g)					
6.48-7.58	15	(0.24±0.01)^a^	(32.8±3.5)^a^	<0.01	<0.05
7.59-8.68	10	(0.23±0.02)^a^	(22.8±2.1)^b^		
8.69-9.78	17	(0.18±0.01)^b^	(20.8±2.0)^b^		
*m*(casein)/*m*(fat)					
0.49-0.55	12	(0.18±0.01)^a^	(25.2±5.0)^a^	<0.05	<0.05
0.56-0.61	16	(0.21±0.01)^ab^	(31.6±3.7)^ab^		
0.62-0.67	14	(0.25±0.01)^b^	(32.9±3.0)^b^		
*w*(Ca^2+)^/(mg/100 g)					
8.12-10.73	13	(0.21±0.01)^a^	31.5±4.0	<0.05	n.s.
10.74-13.34	12	(0.26±0.01)^b^	37.1±3.1		
13.35-15.96	17	(0.24±0.02)^a^	34.4±6.5		
γ(urea)/(mg/100 mL)					
16.25-24.73	14	0.22±0.01	26.3±3.4	n.s.	n.s.
24.74-33.21	10	0.24±0.01	29.1±3.1		
33.22-41.69	18	0.22±0.01	36.4±3.6		
*N*(SC)/(cell/mL)					
<150	12	0.22±0.01	28.5±3.8	n.s.	n.s.
151-600	16	0.23±0.01	25.7±3.6		
601-1.200	14	0.23±0.01	25.7±3.8		

Data are expressed as mean value±standard error. Mean values within a column with different letter in superscript differ significantly (p<0.05), n.s.=not significant, *N*=number of samples, *N*(SC)=somatic cell count, an indicator of milk quality concerning harmful bacteria

The results of this study indicate that the increase in fat content in sheep’s milk significantly (p<0.01) decreased the strength of the obtained gels (Table 3). A marked decrease of gel strength was observed in milk with fat content in the range from 8.69 to 9.78 g/100 g. There was also a low (R=–0.26) but significantly (p<0.01) negative correlation between fat content in sheep’s milk and gel strength (Table 2). Jaramillo *et al.* (*24*) also reported a negative relationship between these parameters but with higher value of coefficient of correlation. On the basis of previous reports (*1*, *23*), this leads to the conclusion that penetration of gel using a cylinder probe caused distortion and rupture of occluded fat globules, releasing liquid and semi-liquid fatty acids, which contributed to lubrication and hence reduction of friction forces on contact surfaces within the paracasein network. Some authors reported positive (*12*, *13*) or little effect (*25*) of fat content in sheep’s milk on gel strength. The increase in milk fat content in sheep’s milk significantly (p<0.05) inhibited syneresis of the obtained gels (Table 3). The lowest syneresis ability was found in gels obtained from milk whose fat content was above 7.59 g/100 g. Results in Table 2 show that fat content in sheep’s milk was significantly (p<0.01) inversely associated with syneresis (R=–0.32) of gels. A similar negative relationship between these parameters was published by Storry *et al.* (*13*) and Jaramillo *et al.* (*24*). As an explanation of these results, Storry *et al.* (*13*) reported that increased fat content in milk leads to an increase in the number of interstices within the gel which are occupied by fat globules and thus increases impedance of whey drainage.

Increasing casein to fat mass ratio in sheep’s milk significantly (p<0.05) enhanced both gel strength and syneresis (Table 3), which is in agreement with the findings of Grandison *et al.* (*26*). Additionally, both gel strength and syneresis had a very low but significant (p<0.01) correlation with casein to fat mass ratio in sheep’s milk (Table 2). This is not in agreement with the results reported by Storry *et al.* (*13*) because they did not find a relationship between casein to fat mass ratio in sheep’s milk and both gel strength and syneresis ability. Since the casein and fat contents in milk tend to change in parallel, they have off-setting effects on gel strength and syneresis (*1*). According to Grandison *et al.* (*26*) gel strength is strongly related to casein content in milk, so strength is expected to be higher for gels obtained from milk with higher casein to fat ratio. As the gel became firmer, channels in the curd could stay open, resulting in effective whey drainage (*23*). On the other hand, increase in casein in relation to fat content tended to reduce clogging effect of fat globules entrapped within the gel.

Our results show that urea concentration in sheep’s milk had no effect on gel strength (Table 3), which is in accordance with the results obtained on cow’s milk published by Martin *et al.* (*27*). Besides, urea concentration in sheep’s milk had no significant effect on the syneresis of rennet curd. However, Abilleira *et al.* (*28*) reported significantly negative correlation between non-protein nitrogen content in sheep’s milk and coagulum firmness. After reviewing available literature, we did not find data about the effect of urea concentration in sheep’s milk on the syneresis ability of rennet curd.

The increase in ionic calcium mass fraction in sheep’s milk had a significant (p<0.05) effect on gel strength. As seen in Table 3, gel strength tends to increase with increasing ionic calcium mass fraction in milk within the range from 8.12 to 13.34 mg/100 g. However, gels obtained from milk samples whose calcium mass fraction was within the range from 13.35 to 15.96 mg/100 g showed lower strength value. Based on this finding, we conclude that the ionic calcium mass fraction and gel strength increase in parallel until they reach the level of availability of binding sites on the casein micelle surface for formation of calcium bridges among paracasein strands. On the other hand, positively charged calcium ions increased the positive charge on the casein micelle surface causing charge repulsion, weaker gel strength (*9*) and less effective syneresis. Concerning the aforementioned fact, Daviau *et al.* (*23*) supposed that less effective syneresis was caused by tortuous and closer channels within softer curd. Apart from the number of cross-links formed among ionic calcium and rennet-altered micelles, it is important to take into consideration that gel strength also depends on insoluble calcium as a key bridging material among casein molecules within casein micelle and consequently on the pH of milk (*6*, *9*). Since the minimum and maximum natural pH values of milk in this study were 6.54 and 6.84, respectively (data not shown), there was a very small loss of insoluble calcium from casein micelle (*9*). In accordance with the results of this study, Bencini (*25*) reported a similar trend for strength of gels obtained from milk with increased ionic calcium mass fraction. This cannot be fully compared with our results since an increase of ionic calcium mass fraction in milk used in their studies was achieved by the addition of calcium chloride, while we used sheep’s milk with natural pH and ionic calcium mass fraction. As reported earlier, addition of calcium chloride reduces pH and increases ionic calcium mass fraction in milk, as well as the mass fraction of insoluble calcium in casein micelle (*6*), which causes their overlapping effect on gel strength. In regards to the results of our study, some authors reported either no (*5*) or weak relationship (*29*) between the free calcium ions in sheep’s milk and gel strength. Tsioulpas *et al.* (*30*) reported that milk with higher amounts of free calcium ions produced a firmer gel. Ionic calcium mass fraction in sheep’s milk did not affect syneresis ability of the obtained gels (Table 3). Although changes in syneresis with increasing ionic calcium mass fraction in sheep’s milk did not reach a statistically significant level, they show a pattern similar to that seen for gel strength (Table 3). These results were also supported by the fact that syneresis was significantly (R=0.20; p<0.05) correlated with gel strength (Table 2). Increasing ionic calcium mass fraction in milk leads to a decrease or increase of whey expulsion (*1*) of the obtained gels, which is not in agreement with our results.

Gel strength remained unchanged regardless of somatic cell count in sheep’s milk (Table 3), which was consistent with the results of Pellegrini *et al.* (*12*). Besides, syneresis decreased within the second group and remained relatively constant within the third group of somatic cell count. Among the numerous studies that have been published concerning the effect of somatic cell count on gel properties, most of them reported a deterioration of its consistency and syneresis (*1*).

At the end of the discussion, it is important to emphasize that although significant correlations (p<0.01; p<0.05) between milk components and gel strength as well as syneresis were observed, these coefficients were low and never higher than 0.35 (Table 2), which was consistent with the results of Bland *et al.* (*5*). Similar results but with slightly higher values of correlation coefficients were reported by Pellegrini *et al.* (*12*).

## CONCLUSIONS

The results of this study show that total solids, casein and fat contents as well as ionic calcium mass fraction of sheep’s milk markedly increased during lactation. However, as lactation progressed, there was an increase in the strength and syneresis of the obtained gels, but correlation between these parameters was weak. Increasing casein to fat mass ratio in sheep’s milk led to the formation of gel with higher strength and syneresis values, while increasing fat content showed the opposite trend. Ionic calcium mass fraction had an effect on gel strength, but not syneresis. Since strength and syneresis of rennet-induced milk gel are important factors in the cheese making, these results could potentially enable the cheese industry to increase cheese production cost-effectiveness. Additionally, the results of this research show that the influence of lactation should not be ignored in the cheesemaking process.
